# Publisher Correction: Modeling group heteroscedasticity in single-cell RNA-seq pseudo-bulk data

**DOI:** 10.1186/s13059-023-02965-2

**Published:** 2023-05-12

**Authors:** Yue You, Xueyi Dong, Yong Kiat Wee, Mhairi J. Maxwell, Monther Alhamdoosh, Gordon K. Smyth, Peter F. Hickey, Matthew E. Ritchie, Charity W. Law

**Affiliations:** 1 Epigenetics and Development Division, The Walter and Eliza Hall Institute of Medical Research, 1G Royal Parade, Parkville, Australia; 2 Department of Medical Biology, The University of Melbourne, Parkville, Australia; 3 CSL Limited, Parkville, Australia; 4 Bioinformatics Division, The Walter and Eliza Hall Institute of Medical Research, 1G Royal Parade, Parkville, Australia; 5 School of Mathematics and Statistics, The University of Melbourne, Parkville, Australia; 6 Advanced Technology and Biology Division, The Walter and Eliza Hall Institute of Medical Research, 1G Royal Parade, Parkville, Australia

**Correction: Genome Biol 24, 107 (2023)**

**https://doi.org/10.1186/s13059-023-02949-2**

Following publication of the original article [1], the authors identified a typesetting error. Authors Yue You, Matthew E. Ritchie and Charity W. Law share co-correspondence for this article. The affiliation for Xueyi Dong has also been corrected. The author group in this correction article has been updated and the original article [1] has been corrected.
